# Actinomycin V Induces Apoptosis Associated with Mitochondrial and PI3K/AKT Pathways in Human CRC Cells

**DOI:** 10.3390/md19110599

**Published:** 2021-10-22

**Authors:** Shiqing Jiang, E Zhang, Hang Ruan, Jiahui Ma, Xingming Zhao, Yaoyao Zhu, Xiaoyu Xie, Ningning Han, Jianjiang Li, Hao Zhang, Weidong Xie, Xia Li

**Affiliations:** 1 Department of Marine Sciences, Marine College, Shandong University, Weihai 264209, China; jiangshiqing@mail.sdu.edu.cn (S.J.); ezhang@mail.sdu.edu.cn (E.Z.); ruanhang@mail.sdu.edu.cn (H.R.); majiahui@mail.sdu.edu.cn (J.M.); zhaoxingming@mail.sdu.edu.cn (X.Z.); zhuyaoyao@mail.sdu.edu.cn (Y.Z.); xiaoyuxie@mail.sdu.edu.cn (X.X.); hanningning@mail.sdu.edu.cn (N.H.); lijianjiang@mail.sdu.edu.cn (J.L.); Zhanghao123@mail.sdu.edu.cn (H.Z.); wdxie@sdu.edu.cn (W.X.); 2 Department of Medicinal Chemistry, School of Pharmaceutical Sciences, Shandong University, Jinan 250012, China

**Keywords:** Act V, CRC, PI3K/AKT, apoptosis, mitochondrial

## Abstract

Actinomycin (Act) V, an analogue of Act D, presented stronger antitumor activity and less hepatorenal toxicity than Act D in our previous studies, which is worthy of further investigation. We hereby report that Act V induces apoptosis via mitochondrial and PI3K/AKT pathways in colorectal cancer (CRC) cells. Act V-induced apoptosis was characterized by mitochondrial dysfunction, with loss of mitochondria membrane potential (MMP) and cytochrome c release, which then activated cleaved caspase-9, cleaved caspase-3, and cleaved PARP, revealing that it was related to the mitochondrial pathway, and the apoptotic trendency can be reversed by caspase inhibitor Z-VAD-FMK. Furthermore, we proved that Act V significantly inhibited PI3K/AKT signalling in HCT-116 cells using cell experiments in vitro, and it also presented a potential targeted PI3Kα inhibition using computer docking models. Further elucidation revealed that it exhibited a 28-fold greater potency than the PI3K inhibitor LY294002 on PI3K inhibition efficacy. Taken together, Act V, as a superior potential replacement of Act D, is a potential candidate for inhibiting the PI3K/AKT pathway and is worthy of more pre-clinical studies in the therapy of CRC.

## 1. Introduction

Colorectal cancer (CRC) is the second most malignant cancer in terms of mortality and diagnosis. Deaths from CRC are expected to be significant, considering the 53,200 estimated deaths in American alone in 2020 [1]. Therefore, an effective therapeutic strategy is long-awaited for CRC patients, especially agents with high efficacy and low toxicity. Actinomycin (Act) V is obtained from *Streptomyces* sp. in the sea and has a similar chemical structure similar to Act D, a well-known drug that has been used to treat multiple human cancers since the 1960s [2,3]. Our previous studies indicated that Act V induces apoptosis in human breast cancer cell lines and suppressed the process of Epithelial–Mesenchymal Transition (EMT) progression, which is important for cancer migration and invasion [4]. It also induced apoptosis in a p53-mediated manner in the human non-small-cell lung cancer cell line A549 [5]. Further studies proved that Act V presented considerably stronger efficacy for cancer cells and less hepatorenal toxicity compared with Act D in vitro and in vivo, making Act V a more potent candidate to replace Act D [6]. Moreover, supercritical fluid chromatography has been applied to isolate Act V from Act D with higher efficacy and less time, making Act V a more accessible and economical drug candidate [7]. While Act V exerts a considerably better efficacy than Act D in human CRC cell lines, identifying its signalling pathway would not only help obtain a better understanding of its function but also provide pre-clinical information.

Mitochondria have an important role in cell energy due to its function of producing adenosine triphosphate (ATP), and it is also vital in apoptosis-related cell death [8,9]. In the field of cancer, chemotherapy is thought to be an effective way to induce apoptosis; hence, an understanding of mitochondria may lead to novel treatment strategies. In general, once mitochondria are activated by upstream signals, the mitochondrial membrane potential (MMP) decreases, and it becomes easier for the mitochondrial outer membrane (MOM) to release cellular cytochrome c as well other proteins that play a vital role in activating the downstream caspase cascade [10]. Thus, the cell starts apoptosis, and an apoptosis-related caspase cascade reaction occurs. The Bcl-2/Bax family exists in mitochondrial membranes involved in apoptosis regulation. Bcl-2 and Bcl-XL inhibit apoptosis, and these anti-apoptotic proteins are usually found over-expressed in tumors, which may lead to resistance to apoptosis [11]. Bax protein can induce cytochrome c release and can be inhibited by Bcl-2 family proteins [12].

The phosphoinositide 3-kinase (PI3K)-AKT signaling pathway is widely acknowledged as a most commonly elevated one in human cancers, including CRC, and it is essential in cell survival and growth as well as in glucose metabolism and anabolic metabolism [13,14]. Aberrant PI3K activity brings about upregulation of AKT family proteins; in fact, phosphorylated AKT (p-AKT) is often a hallmark of PI3K activation [15]. In CRCs, statistics show that 14% of cases carried PIK3CA catalytic domain mutations, which is known to activated PI3K signaling [16,17]. In 2019, the U.S. FDA approved the first PI3K inhibitor in cancer treatment [18]. Thus, the PI3K inhibitors may enable a reduction in the abnormal proliferation and show promise as a potential drug in CRC diseases.

Meanwhile, aberrant PI3K/AKT signaling affects not only cell apoptosis through mitochondrial outer membrane permeabilization (MOMP) but also the key protein AKT, which controls cell proliferation and inhibition [19,20]. AKT can directly phosphorylate the pro-apoptotic Bcl-2 family proteins Bax, Bim, and Bad and caspase-9 [21,22,23]. Thus, PI3K inhibition may trigger apoptosis in a highly synergistic manner similar to that of mitochondrial apoptosis. Several research studies have already proved that the inhibition of PI3K results in the sensitization of chemotherapy [19,24].

Act D is a first-line chemotherapeutic agent in the clinic, but its high toxicity severely limits its use. In our previous studies, Act V exhibited strong anticancer activities and less hepatorenal toxicity compared with Act D, which highlights the possibility of the replacement of Act D with a superior agent and makes further investigations worthwhile. In this study, we aim to further evaluate the pharmacological features of Act V in the CRC HCT-116 cell line. To investigate the cellular mechanism underlying its anticancer activity, we assessed the impact of Act V on the PI3K/AKT pathway and the mitochondrial apoptotic pathway, and the two pathways can exert synergistic apoptotic effects.

## 2. Results

### 2.1. Act V Inhibited Human CRC Cell Lines

**Figure 1:**
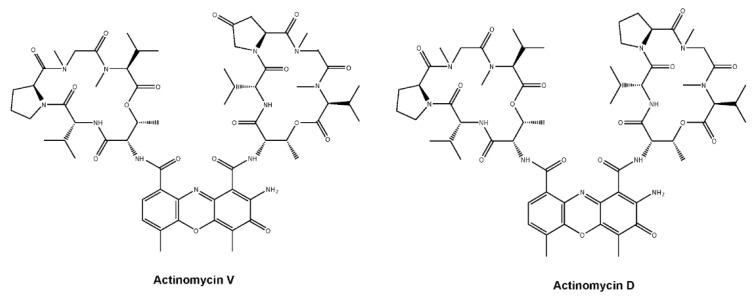
Structures of Act V and Act D.

**Figure 2:**
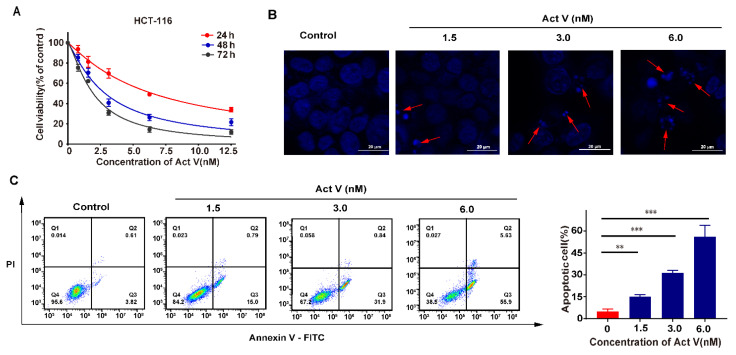
Act V suppresses cell viability and induces apoptosis in CRC. (**A**) Act V treatment inhibited the cell viability of HCT-116 cells in a time-dependent manner; (**B**) Fluorescence images of HCT-116 cells after 24 h of Act V treatment by DAPI staining; (**C**) HCT-116 apoptotic cells after 24h Act V treatment, detected by flow cytometry. Scale bars = 20 μm. ** *p* < 0.01, *** *p* < 0.001.

After human colorectal cell lines (HT-29, HCT-116, SW620, and SW480) were incubated for 24 h, a colorimetric MTT assay was performed to evaluate the effect of Act V and Act D (Figure 1). Act V inhibited the proliferation of human colorectal cell lines in vitro and had the strongest effect on the HCT-116 cell line, with an IC_50_ for 48 h of 2.85 ± 0.10 nM. The IC_50_ values for Act V (48 h treatment) on other CRC cell lines were also drastically small compared with Act D: 6.38 ± 0.46 nM to HT-29, 6.43 ± 0.16 nM to SW620 and 8.65 ± 0.31 nM to SW480 (Table 1). Human normal kidney cell line HEK-293T and human normal liver cell line QSG-7701 are also under test by an MTT assay, taking their IC_50_ values (68.3 ± 1.2 nM to QSG-7701 and 82.6 ± 0.9 nM to HEK-293T) into consideration, Act V exerts a strong effect on human colorectal cell lines and a relatively weak effect on normal liver and kidney cells Further MTT assays showed that Act V suppressed the proliferation of HCT-116 cell lines in a time-dependent way (Figure 2A).

### 2.2. Act V Induced Apoptosis in the CRC Cell Line HCT-116

To investigate the apoptotic effect of Act V, we chose the HCT-116 cell line as the experimental cell line. The cell morphological assessment of nucleus changes was determined by DAPI staining using different concentrations of Act V for 24 h. Under the fluorescence microscope, we observed a homogeneous distribution of chromatin in the nucleus and membrane blebbing, and apoptotic body in the treated groups. At concentrations of 1.5 nM and 3.0 nM, apoptotic bodies could be observed, and at concentration of 6.0 nM, significant apoptosis was induced (Figure 2B).

To determine the specific proportion of apoptotic cells, cells were stained by Annexin V-FITC and propidium iodide (PI) using an apoptosis detection kit. The apoptotic cells were illustrated in the Q2 and Q3 area by flow cytometry (Figure 2C). The data show that the percentage of apoptotic cells increased dramatically after treatment with Act V, from 4.43%, 15.79%, and 32.74% to 61.53% by the sequence of different concentrations of Act V. These results indicated that Act V might induce cell death by inducing apoptosis.

### 2.3. Act V Decreased Mitochondrial Membrane Potential (MMP) in the CRC Cell Line HCT-116

**Figure 3:**
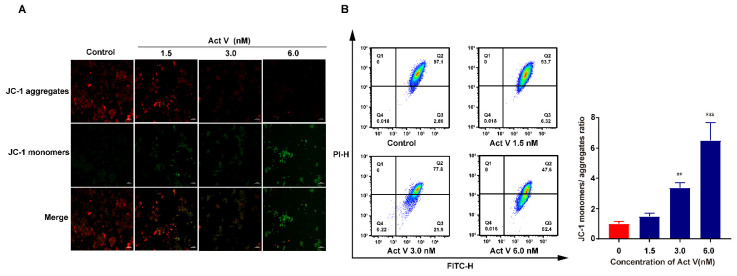
Effects of Act V on mitochondrial membrane potential. (**A**) HCT-116 cells were treated with varying concentrations of Act V for 24 h and then treated with a JC-1 probe and observed under a fluorescence microscope. The representative images for each condition are shown. Scale bars = 100 μm. Red fluorescence represents JC-1 aggregates, and green fluorescence represents JC-1 monomers. (**B**) HCT-116 cells were treated with varying concentrations of Act V for 24 h and then treated with a JC-1 probe and detected by flow cytometry; the JC-1 monomers/JC-1 aggregate ratio was calculated. ** *p* < 0.01, *** *p* < 0.001.

Mitochondrial dysfunction can result in apoptosis, and we investigated whether mitochondrial dysfunction occurred through changes in the MMP (∆Ψm) using JC-1 staining, as a decrease in ∆Ψm is a hallmark of early apoptosis [25]. In healthy mitochondria, JC-1 accumulated in the matrix, which revealed red fluorescence; in contrast, JC-1 stayed as monomers in the damaged mitochondria and revealed green fluorescence. Figure 3A shows that Act V decreased ∆Ψm in a dose-dependent manner; therefore, it may result in mitochondrial dysfunction.

To better understand the ratio of JC-1 monomers to JC-1 aggregates, we performed a JC-1 flow cytometry assay. As the concentration increased, the ratio climbed to 1.49-, 3.38-, and eventually 6.5-fold of the control (Figure 3B). These results revealed that Act V decreased the concentration of MMP in the HCT-116 cell line.

### 2.4. The Effect of Act V on the Expression of Proteins Related to Mitochondrial Apoptotic Pathways in HCT-116 Cells

**Figure 4:**
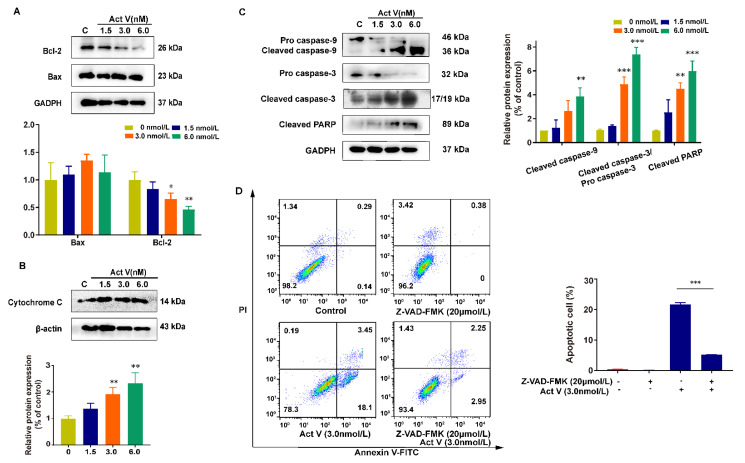
The effect of Act V on the expression of mitochondrial apoptotic pathway-related proteins. (**A**–**C**) Western blot analysis result of the expression levels of Bax, Bcl-2, cytochrome c, caspase-9, pro caspase-3, cleaved caspase-3, and cleaved PARP. (**D**) The apoptotic cell proportion was investigated using Act V and the caspase inhibitor Z-VAD-FMK: HCT-116 cells treated with Z-VAD-FMK and Act V induced a dramatically low apoptotic cells proportion compared with Act V alone. * *p* < 0.05, ** *p* < 0.01, *** *p* < 0.001.

To determine the underlying mechanisms, mitochondrial apoptotic pathway-related proteins were investigated by Western blotting. Bcl-2 family proteins control the balance of cell life and death, and apoptosis in mitochondrial dysfunction. From Figure 4A, we can learn that, as the concentration increases, the expression of Bax/Bcl-2 increases. The increased Bax/Bcl-2 then causes MOMP, leading to the release of cytochrome c from the mitochondria to the cytoplasm. Thus, we can see that the cytoplasmic cytochrome c increases dramatically (Figure 4B). Moreover, the caspase cascade is also activated, so the expression of cleaved caspase-9, cleaved caspase-3, and cleaved poly-ADP ribose polymerases (PARP) increases sequentially, leading to cell death [26] (Figure 4C).

We then used Z-VAD-FMK (a broad-spectrum caspase inhibitor) in the negative control group and the combination group to further prove that Act V induces apoptosis in a mitochondrial manner, and this antitumor effect can be reversed by a caspase inhibitor, considering that the apoptotic cell proportion dropped from 21.6% to 5.2% (Figure 4D).

Together, these results suggest that Act V induced apoptosis is associated with the mitochondrial apoptotic pathway.

### 2.5. Act V Regulates the PI3K/AKT Pathway

**Figure 5:**
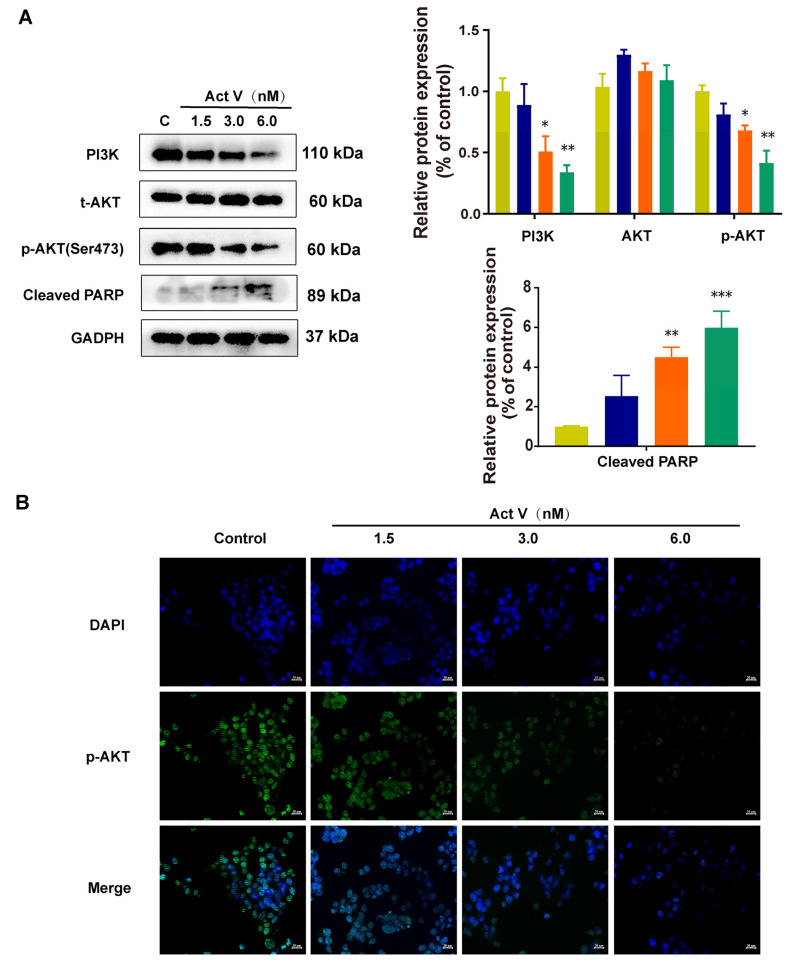
Effect of Act V against the PI3K/AKT pathway in HCT-116 cells. (**A**) Western blot analysis was used to investigate the effects of Act V on PI3K, AKT, p-AKT, and cleaved PARP proteins. (**B**) Immunofluorescence staining was used to investigate the expression of p-AKT; images are shown separately; DAPI (blue) and p-AKT (green). Scale bars = 20 µm. * *p* < 0.05, ** *p* < 0.01, *** *p* < 0.001.

The PI3K/AKT pathway is grossly elevated in cancer and acts as a hallmark [27]. AKT, a vital oncogenic effector in this pathway, is tightly linked with PI3K. To further study the underlying mechanism of Act V on apoptosis, the expression of PI3K/AKT signaling was investigated in HCT-116 cells. HCT-116 cells were treated with 0 nM, 1.5 nM, 3.0 nM, and 6.0 nM of Act V for 24 h, and the related proteins were analyzed by Western blotting. From Figure 5A, we can see that the expressions of PI3K, p-AKT, and cleaved PARP were decreased after treatment. In conclusion, Act V may induce apoptosis through the PI3K/AKT pathway in HCT-116 cells. Moreover, immunofluorescence staining revealed that p-AKT was mostly in the nucleus and was suppressed after Act V treatment (Figure 5B).

### 2.6. Inhibition of PI3K Proves the Effect of Act V on the PI3K/AKT Pathway

**Figure 6:**
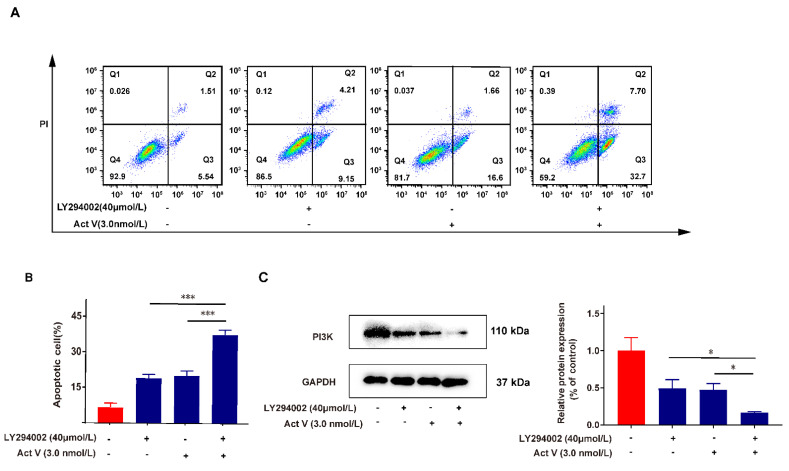
Act V exerts a parallel apoptotic effect to the PI3K inhibitor LY294002. (**A**) Flow cytometry detected the apoptotic cell proportion after HCT-116 cells were inoculated and treated with Act V, LY294002, or a dual treatment. (**B**) The proportion of apoptotic cells was statistically recorded. (**C**) The PI3K expression was investigated using Act V and the PI3K inhibitor LY294002: HCT-116 cells treated with dual agents expressed a dramatically low level of PI3K protein compared with the single agent alone. * *p* < 0.05, *** *p* < 0.001.

LY294002 (LY) is a PI3K inhibitor that can inhibit PI3K. We used 40 μM LY treatment in the positive control group and the enhanced group to further prove that the PI3K/AKT pathway is involved in Act V’s antitumor effects. Figure 6A and Figure 6B reveal that the 40 μM LY treatment increased the apoptotic cells from 7.05% (control group) to 13.36% and that the enhanced group (co-cultured with 40 μM LY and 3.0 nM Act V) augmented the portion of apoptotic cells to 40.4%. These data confirmed that the apoptotic effect of Act V was consistent with LY, and a combination treatment further exaggerated the pro-apoptotic effect. Further Western blot experiments on the PI3K expression also revealed that the expression of PI3K dramatically decreased when co-treated with the PI3K inhibitor LY and Act V compared with Act V or LY alone. The results showed that Act V inhibited PI3K, which is consistent with LY (Figure 6C).

### 2.7. Molecular Docking Analysis of Act V against PI3K

**Figure 7:**
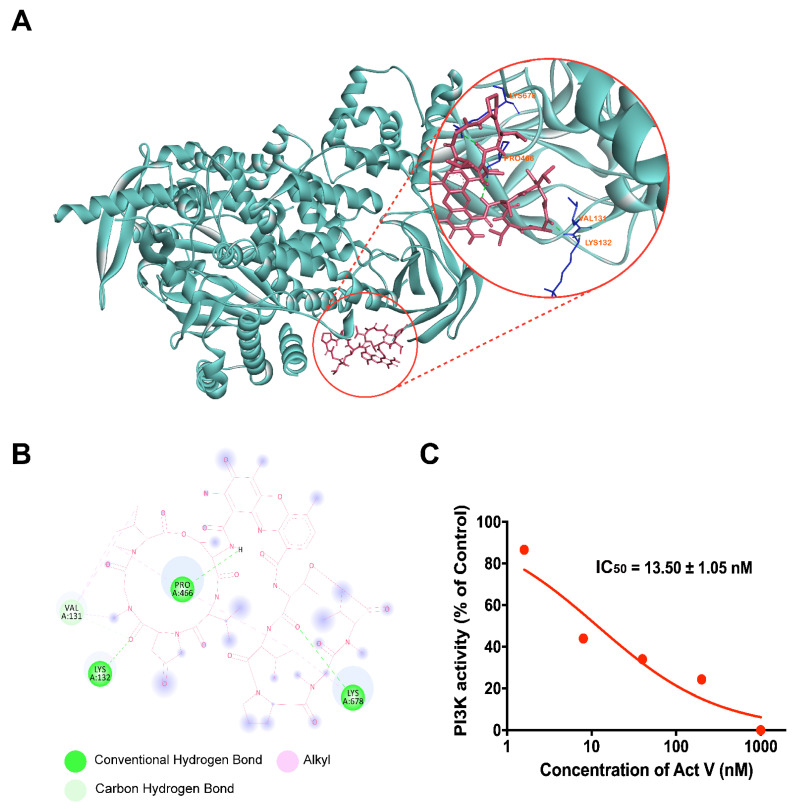
PI3K inhibitory effect of Act V investigated by Auto-DockTools software, Discovery studios, and HTRFKinEASE-TK kit. (**A**) Auto-DockTools software and discovery studios were employed to obtain the optimal docking model of Act V to the PI3K protein. The optimal binding configuration is shown, with a −7.39 KJ/mol binding energy. (The PI3Kα protein model was taken from Protein Data Bank, ID: 3ZIM.) (**B**) A simplified 2D interaction model is illustrated, with conventional hydrogen bonds, carbon bonds, and alkyl interaction shown. (**C**) The HTRF kit was used to analyze the inhibition efficacy of Act V. The data are presented as the mean ± SD (standard deviation) of triplicate independent examinations.

Molecular docking simulations were carried out using AutoDockTools-1.5.6 to ensure the binding affinity of PI3K and Act V. Hydrogen bonds and the lowest binding energy are considered when selecting the optimal interaction configuration. As PI3K γ/δ only exists in immune cells and PI3Kα is usually mutated in CRC, we selected PI3Kα. According to Figure 7A, we can see the optimal interaction binding mode of PI3Kα and Act V. A molecular docking analysis shows that the lowest binding energy of Act V and PI3Kα was −7.39 KJ/mol. Thus, we can predict that Act V inhibits the PI3K/AKT pathway by binding to PI3Kα, and it exerts a targeted binding potential.

### 2.8. Inhibitory Activity of Act V against PI3K Using Homogeneous Time-Resolved Fluorescence Technology (HTRF^®^)

HTRF^®^ is acknowledged as a reliable assay to measure the blockade effect of chemicals against specific proteins in vitro. The results are shown in Figure 7B and Table 2. Act V presents a 28-fold greater potency than the PI3K inhibitor LY (13.50 nM versus 390.97 nM). These results showed that Act V might be a more potent PI3K inhibitor, leading to the potential for further research in CRC therapy by inhibiting PI3K.

## 3. Discussion

For decades, anti-cancer compounds have been focused on natural origin and most are from the terrestrial ecosystem; however, the marine ecosystem constitutes 80% biodiversity, making it a fertile land for anti-cancer compounds [28]. Act D, an old drug approved in the 1960s, was used to treat multiple diseases including Wilms’ tumor and solid tumors [29]. Colorectal cancer is often considered a common solid tumor worldwide with a high mortality rate awaiting more drug insights. Our previous studies reported that Act V, a marine-derived compound, inhibited the EMT process in human breast cancer cell lines, in which the suppression of snail and slug proteins was involved in the mechanism [4]. Additionally, in the human non-small-cell lung cancer cell line A549, Act V functions in the p53-mediated mechanism [5]. Moreover, in this study, we report that Act V exerts a greater inhibitory effect in the human CRC HCT-116 cell line than Act D, inducing apoptosis associated with the mitochondrial apoptotic pathway and the PI3K/AKT pathway synergistically, which enhances our comprehension of Act V’s mechanism and provides more detailed preclinical research data.

An elucidation of the mechanism revealed that Act V induced apoptosis associated with the mitochondrial apoptotic pathway in HCT-116 cells. During the apoptosis process, DAPI staining revealed representative morphological changes in the nucleus. The results showed that Act V remarkably decreased ΔΨm; increased cytoplasmic cytochrome c expression; and increased the expressions of cleaved caspase-9, cleaved caspase-3, and PARP, indicating that Act V induces apoptosis in HCT-116 cells via the mitochondrial apoptosis pathway.

PI3Ks are divided into three classes in humans, and class I PI3Ks directly function on signal transduction pathways [30]. Among the class I PI3Ks, PI3Kα and PI3Kβ are expressed ubiquitously, while PI3Kγ and PI3Kδ are usually found in immune cells or hemocytes [30]. Thus, PI3Kα and PI3Kβ are of great interest as a target in solid tumors. Moreover, the PI3K/AKT pathway has a synergetic effect in contributing to an increased MOMP, which impacts protein release from the intermembrane and acts as a key segment of apoptosis [19,23]. The results showed that Act V remarkably decreased the expression of PI3K and p-AKT, and it exerts a strong efficacy in PI3K inhibition in a much greater way than the recognized PI3K inhibitor LY294002, with an IC_50_ of 13.50 nM versus 390.97 nM. In addition, an interaction between Act V and PI3Kα was investigated via molecular docking analysis, strongly supporting that Act V may have a targeted potential on PI3Kα inhibition. Although PI3K inhibitors still face several problems, such as low tolerance and drug resistance, insights into the isoform-selective PI3K inhibitors and combination therapies have fortunately drawn attention due to the selection of patients harboring different mutations and the optimization of schedules of combinations to ameliorate toxicity [31,32].

In conclusion, this study showed that Act V-induced apoptosis in HCT-116 cells is associated with mitochondrial and PI3K/AKT pathways. Act V is a promising candidate for further research as a superior potential replacement of Act D in CRC. Although it has a strong function in inhibiting PI3K with a tiny dose in vitro, caution should still be taken when considering developing it into CRC therapies, particularly with respect to toxicity, drug resistance, combination treatments, and specific PI3K isoforms. Further investigation will be needed to determine which other specific isoforms of PI3K were inhibited and what are their intermolecular interactions. Animal experiments are also needed to compare its internal effect with classic chemotherapeutic agents in the clinic as well as its potential use in combination therapies to treat CRC.

## 4. Materials and Methods

### 4.1. Chemical and Reagents

Act V (purity > 98%) and Act D (purity > 98%) were obtained from Dr. Xie (Shandong University, Weihai, China), isolated from *Streptomyces* sp. [33]. The compounds were dissolved in dimethyl sulfoxide (DMSO). Antibodies against AKT, PI3K, Bcl-2, Bax cleaved caspase-9, and cleaved caspase-3 were purchased from Cell Signalling Technology (CST Inc., Beverly, MA, USA). Antibodies against p-AKT, cytochrome C, and PARP were purchased from WanLei Biotechnology (Shenyang, China). Antibodies against GAPDH and β-Actin were purchased from Beyotime Institute of Biotechnology (Shanghai, China). The Annexin V-FITC apoptotic detection kit, JC-1 (“5,5′,6,6′-tetrachloro-1,1′,3,3′-tetraethyl-imidacarbocyanine iodide”) staining kit, LY294002 (PI3K inhibitor), DAPI staining solution, and cell mitochondrial isolation kit were purchased from Beyotime Institute of Biotechnology (Shanghai, China). The HTRFKinEASE-TK kit was purchased from Cisbio (Cisbio Bioassays Co., Codolet, France). The PI3K antibody in the kinase inhibition assay was obtained from Abcam, Inc. (Cambridge, MA, USA). All chemicals used in this study were commercial reagent grade products.

### 4.2. Cell Lines and Cell Culture

Human CRC cell lines HCT-116, HT-29, SW480, SW620, normal human liver cell line LO-2, and normal human kidney cell line HEK-293T were purchased from the Shanghai Institute for Biological Sciences (SIBS), Chinese Academy of Sciences (Shanghai, China). All cells were cultured in RPMI 1640 medium (Living Biotechnology Co., Ltd., Beijing, China) with 10% fetal bovine serum (Gibico life technologies, CA, USA) and 100 units/mL of penicillin and streptomycin (100 µg/mL). The cells were cultured in an incubator with 5% CO_2_ at 37 °C.

### 4.3. MTT Assay

An MTT assay was used to detect the inhibitory effect of Act V and Act D. The cells were inoculated in 96-well plates overnight and then treated with Act V or Act D in different concentrations for 24-72h. The cells in the control group were treated with 0.1% DMSO. After treatment, the medium was removed and 15 µL 5 g/L MTT (Sigma-Aldrich Corp., St. Louis, MO, USA) was added for a further 4 h at 37 °C. The MTT medium was removed, and 150 µL DMSO was added to each well. The absorbance was measured at 570 nm using a microplate reader. The cell survival rate was calculated against that of the control group. The IC_50_ values were calculated using three separate data sets by Graphpad Prism 9.0 (Graphpad, CA, USA).

### 4.4. Flow Cytometry Analysis

The cells were inoculated in 6-well plates and incubated overnight before being treated with different concentrations of Act V, 40 μM LY294002, or 20 μM Z-VAD-FMK or cotreated with 40 μM LY294002 and Act V, or 20 μM Z-VAD-FMK and Act V for 24 h. The Annexin V–FITC apoptotic detection kit was used to detect the proportion of apoptotic cells, and the samples were detected by BD FACSVia flow cytometry (Becton Dickinson, NJ, USA). The early phase of apoptotic cells was stained by Annexin V-FITC and PI negative (Q3 area), and the late phase of apoptotic cells was stained by Annexin V-FITC and PI positive (Q2 area).

To detect the MMP, the HCT-116 cells were inoculated, treated with different concentrations of Act V, then disassociated, and stained with JC-1 (10 μg/mL) for 30 min at 37 °C. The cells were washed with PBS twice and immediately analyzed by BD FACSVia flow cytometry.

### 4.5. Mitochondrial Membrane Potential Assay

The HCT-116 cells were inoculated, treated with different concentrations of Act V and then stained with JC-1 (10 μg/mL) for 30 min at 37 °C. The cells were washed with PBS twice and immediately observed under the fluorescence microscope.

### 4.6. DAPI Staining

DAPI staining was used to clarify the morphological changes of the nucleus of the treated groups. HCT-116 cells were inoculated on thin glass coverslips in 12-well plates and then treated with different concentrations of Act V for 24 h. The cells were washed with PBS and fixed with 4% paraformaldehyde for 15 min at room temperature before permeabilized treatment with 0.5% Triton X-100 for 20 min and were finally stained with 5 μg/mL DAPI in a darkroom for 15 min. After gentle washing with cold PBS three times, coverslips with cells were prepared as a specimen using a glass slide and 20 µL glycerol. Representatives images of different groups were captured with an inverted fluorescence microscope for further morphological analysis.

### 4.7. Preparation of Cytosolic Protein

A cell mitochondrial isolation kit was used to isolate and collect cell plasma protein. After the cells were seeded in a 6-well plate overnight and treated with different concentrations of Act V for 24 h, the cells were digested, collected in a cytoplasmic extraction buffer for 10 min, and disrupted with a cell breaker. The cell homogenates were centrifuged at 600× *g* and 12,000× *g* at 4 °C for 15 min sequentially. The supernatant protein was collected for further Western blotting analysis.

### 4.8. Western Blot Analysis

A Western blot analysis was used to determine the changes on expressions of specific proteins after Act V treatment. The cells were inoculated and treated with specific concentrations of Act V for 24 h, and the whole protein was obtained using cold RIPA lysis buffer. The protein concentrations were measured with a BCA protein assay kit and boiled with protein loading buffer. The samples were subjected to 10–12% SDS-PAGE and transferred to PVDF membranes. The membranes were blocked in 5% skimmed milk for 2 h at room temperature and incubated in the primary antibody diluent overnight. Then, the membranes were washed with TBS-T buffer and incubated in secondary HRP-conjugated IgG buffer (anti-rabbit or anti-mouse) for 1 h at room temperature accordingly. The proteins were visualized by ECL detection (ECL^®^, Amersham Biosciences, Little Chalfont, UK), and quantified by ImageJ software 1.8.0 (National Institutes of Health Maryland, USA).

### 4.9. Immunofluorescence Staining

HCT-116 cells were inoculated overnight on 12-well plates equipped with a thin cover glass and treated with different concentrations of Act V for 24 h. The cells were washed by PBS twice, then permeabilized in 0.5% Triton X-100 for 15 min, washed twice, incubated in 5% BSA for 1 h, and then washed twice before being sunken into p-AKT antibody overnight at 4 °C. FITC goat anti-rabbit secondary antibody was then used for 1 h at 37 °C before the cells were washed and stained by DAPI for 10 min. Representative images of different groups were captured with an inverted fluorescence microscope for further morphological analysis.

### 4.10. Docking Studies

AutoDockTools-1.5.6 (The Scripps Research Institute, CA, USA) and Discovery studios were used to investigate the molecular docking character. The PI3K 3D structure was downloaded from the Protein Data Bank (PDB code: 3ZIM). AutoDockTools-1.5.6 provides several predicted binding modes by automated docking of the ligand with the target. The conformation with the lowest binding energy was assigned as the most suitable model for chemicals and ligands. The Lamarckian genetic algorithm was used in the docking analysis.

### 4.11. PI3K Inhibition Viability Test by Homogeneous Time-Resolved Fluorescence (HTRF^®^) Analysis In Vitro

The HTRFKinEASE-TK kit was used to analyze the inhibition efficacy of Act V on PI3K and to specify its IC_50_. Besides Act V, there were three control groups: positive control, blank control, and cryptate control. Act V was prepared as a 10 nM solution. TK substrate biotin, ATP and PI3K protein were prepared with kinase buffer at concentrations of 1 μM, 2.93 μM, and 0.02 g/L. According to Cisbio’s protocol, 5 μL of TK substrate biotin and an ATP mixture (1:1 by volume) was placed into a 384-well plate, which was sealed and incubated for 1 h at room temperature. Then, 2 μL of 10 mol/L Act V was added to the treated group. LY instead of Act V was added as the positive control, and the buffer instead of PI3K was added as the negative control. After incubation, a 10 µL mixture of SA-XL665 and TK antibody cryptate (1:1 by volume) was added to end the reaction system. After 1 h at room temperature, the absorbance was measured at 665 nm and 615 nm using a microplate reader compatible with HTRF^®^. IC_50_ values were calculated using three separate data sets by Graphpad Prism 9.0.

### 4.12. Statistical Analysis

All data are presented as mean ± standard deviation from a minimum of three independent experiments. All data were analyzed using a one-way Analysis of Variance (ANOVA) for multiple comparisons by SPSS 16.0 (SPSS Inc., Chicago, IL, USA). * *p* < 0.05 was considered significant.

## Figures and Tables

**Table 1:** IC_50_ of Act V and Act D in different cell lines at 48 h.

Cell Lines	Act V	Act D
IC_50_ (nmol/L)		
**HCT-116**	2.85 ± 0.10	10.24 ± 0.29
**HT-29**	6.38 ± 0.46	15.27 ± 0.42
**SW620**	6.43 ± 0.16	13.41 ± 0.23
**SW480**	8.65 ± 0.31	11.78 ± 0.20
**QSG-7701**	68.3 ± 1.2	66.5 ± 1.1
**HEK-293T**	82.6 ± 0.9	64.2 ± 1.6

IC_50_ of Act V and Act D on HCT-116, HT-29, SW620, SW480, QSG-7701, and HEK-293T cells at 48 h. Cells were inoculated and treated with different concentrations of compounds; then, the IC_50_ values (concentration resulting in 50% inhibition of cell growth) were measured by MTT assay. Data are presented as the mean ± SD (standard deviation) of triplicate independent examinations.

**Table 2:** IC_50_ values of Act V and LY294002 on PI3K inhibition.

Compounds	Act V	LY294002
**IC_50_ (** **nmol/L)**	13.50 ± 1.05	390.97 ± 14.50

The HTRFKinEASE-TK kit was used to analyze the inhibition efficacy of Act V and LY294002 on PI3K and to specify its IC_50_. LY294002 was used as a positive control.

## Data Availability

The data presented in this study are available from the corresponding author upon request.
